# 10 Hz rTMS over right parietal cortex alters sense of agency during self-controlled movements

**DOI:** 10.3389/fnhum.2014.00471

**Published:** 2014-06-25

**Authors:** Anina Ritterband-Rosenbaum, Anke N. Karabanov, Mark S. Christensen, Jens Bo Nielsen

**Affiliations:** ^1^Department of Nutrition, Exercise and Sports, Panum Institite, University of CopenhagenCopenhagen, Denmark; ^2^Department of Neuroscience and Pharmacology, Panum Institite, University of CopenhagenCopenhagen, Denmark; ^3^Danish Research Center for Magnetic Resonance, Copenhagen University Hospital HvidovreHvidovre, Denmark; ^4^Danish Neuroscience Center, Cognitive Neuroscience Research Unit, Aarhus UniversityAarhus, Denmark

**Keywords:** sense of agency (SoA), self-controlled movement, repetitive TMS, inferior parietal cortex (IPC), sensorimotor comparison

## Abstract

A large body of fMRI and lesion-literature has provided evidence that the Inferior Parietal Cortex (IPC) is important for sensorimotor integration and sense of agency (SoA). We used repetitive transcranial magnetic stimulation (rTMS) to explore the role of the IPC during a validated SoA detection task. 12 healthy, right-handed adults were included. The effects of rTMS on subjects' SoA during self-controlled movements were explored. The experiment consisted of 1/3 self-controlled movements and ^2^/_3_ computer manipulated movements that introduced uncertainty as to whether the subjects were agents of an observed movement. Subjects completed three sessions, in which subjects received online rTMS over the right IPC (active condition), over the vertex (CZ) (sham condition) or no TMS but a sound-matched control. We found that rTMS over right IPC significantly altered SoA of the non-perturbed movements. Following IPC stimulation subjects were more likely to experience self-controlled movements as being externally perturbed compared to the control site (*P* = 0.002) and the stimulation-free control (*P* = 0.042). The data support the importance of IPC activation during sensorimotor comparison in order to correctly determine the agent of movements.

## Introduction

Distinguishing one's own actions from actions of others is a key component of social interaction and usually happens effortlessly even in the most complex situations like playing a fourhanded piano piece. The ability to correctly identify self-produced movement is called sense of agency (SoA) and is based on integration of sensory (most often visual and proprioception) and motor information (Gallagher, 2000). An altered sense of agency can occur both in mental illness and following brain injury and can severely impact the ability to control movements and alter self-consciousness (Farrer and Frith, 2002; Ritterband-Rosenbaum et al., 2011).

Neuropsychological evidence and brain imaging data associate the sense of agency with areas in the Inferior Parietal Cortex (IPC) which are generally important for a multitude of complex sensory and motor tasks (e.g., visuo-motor integration, visual attention, spatial representations, reaching and grasping movements, action observation) (Andersen et al., 1987; Culham and Kanwisher, 2001; Culham and Valyear, 2006; Iacoboni, 2006; Rushworth and Taylor, 2006). Neuropsychological data from lesion studies often associate damage in IPC to distortions in self-awareness such as hemi-spatial neglect (unawareness of the visual field and body side contralateral to the lesion) (Mort et al., 2003), asomatognosia (loss of ownership over a limb) (Baier and Karnath, 2008) or alien-limb syndrome (distorted sense of agency over own movements) (Franck et al., 2001; Fourneret et al., 2002). In experimental settings, neurological patients (all with lesions involving the left parietal lobe) also show changes in awareness of voluntary action (Sirigu et al., 2004). However, since brain damage may be functionally more extensive than what can be determined by imaging techniques and usually involve adaptations to compensate for lost functions, it is hard to make spatially precise inferences about the role of the individual cortical areas in agency attribution in the healthy brain from such studies.

Several brain-imaging studies have studied the role of the IPC during agency attribution in healthy individuals (Farrer and Frith, 2002; Farrer et al., 2008; Nahab et al., 2011). Since spontaneous misattributions of agency are rare in healthy participants all these studies use external perturbations of the feedback either temporally or spatially to challenge the agency attribution. These studies consistently show that activation of the IPC and the adjacent areas increase with a subjective loss of agency. In a recent EEG study (Ritterband-Rosenbaum et al. submitted and planned to appear in this issue) we were able to identify an IPC-pre supplementary motor area (preSMA) network, which showed coupled activity when subjects experienced agency over their movements. Results from the study suggest that the IPC supplies the preSMA with information about a mismatch of sensorimotor and visual information after the movement has been performed.

TMS allows that conclusions regarding the causal relationship between a brain region and behavior may be made by producing a transient and localized disruption in normal brain activity (Pascual-Leone et al., 2000). Some previous TMS studies have investigated the role of the IPC and the adjacent parietal areas in temporal and spatial aspects of agency attribution (MacDonald and Paus, 2003; Preston and Newport, 2008). MacDonald and colleagues investigated the temporal assessment of self-controlled movements and showed that participants' awareness of movement onset was disrupted after stimulation of the left superior parietal lobule. Preston et al. investigated the outcome assessment of reaching movements and reported a decreased tendency for self-attribution for spatially perturbed and un-perturbed trials after TMS of the right IPC (Preston and Newport, 2008). However, in that study participants were only able to observe the start and end point of the movement with the largest part of the movement trajectory occluded. A noticeable difference between the imaging literature and some of the brain stimulation results is that whereas imaging work consistently reports increased activity of the IPC with increasing levels of external perturbation (e.g., when participants do not experience agency), the TMS work seems to suggest that disrupting this region modulates agency relatively unspecifically whether the observed movement is externally generated (e.g., a manipulated movement) or not (e.g., a self-controlled movement).

The goal of the present study was to further disentangle the role the IPC has in agency perception during different degrees of spatial feedback perturbations. Since we did not perturb the temporal movement feedback we cannot draw conclusions about the role of the IPC in temporal agency perception. We used a validated arm-reaching paradigm (Ritterband-Rosenbaum et al., 2011, 2012). In two thirds of trials different levels of spatial perturbation (10 and 15°) were added to introduce uncertainty as to whether the subjects were the agent of the observed movement or not. Participants performed three different sessions during which online rTMS (rTMS) was given over the right IPC or over the vertex. In the third session a sound-matched, stimulation-free control was applied.

Imaging studies consistently report that activity in the IPC decreases in trials with high-perceived agency, rTMS, on the other hand is assumed to be state-dependent and may influence less active neural populations most strongly (Silvanto and Pascual-Leone, 2008; Silvanto et al., 2008). This is why we hypothesize that the self-controlled movements might be most susceptible to rTMS, since in this condition there is most scope for the IPC firing rate to be increased by stimulation. On a first glance, this seems counterintuitive but the notion of a higher degree of firing rate modulability might offer a neural basis for the state-dependency phenomena. The idea is also consistent with clinical observations reporting that normal brain activity can interfere with the spread of an epileptic discharge (Wilkins et al., 2004).

## Materials and methods

### Population

Fourteen healthy, naïve, right-handed adults (mean age: 25.6 ± 6.7 years, SD, 5 males) participated in the study. None of the participants had a history of neurological or psychiatric disorders. None of them had metal implants. Handedness was assessed prior to the experiment using the Edinburgh Inventory questionnaire (Oldfield, 1971). All participants in the study had an anatomical MRI scan made within the past 2 years. One participant had to be excluded due to failure to perform the task properly and one withdrew after completing 2 of the 3 sessions because of discomfort with the TMS; therefore, only 12 participants were included in further analysis. Subject selection and all TMS procedures were in accordance with the TMS safety guide lines (Rossi et al., 2009). Written informed consent was obtained for each subject prior to the experiment. The study was conducted according to the Helsinki declaration and was approved by the local ethics committee in Copenhagen, Denmark (protocol number: H-A-2008-029).

### General procedure

**Figure 1:**
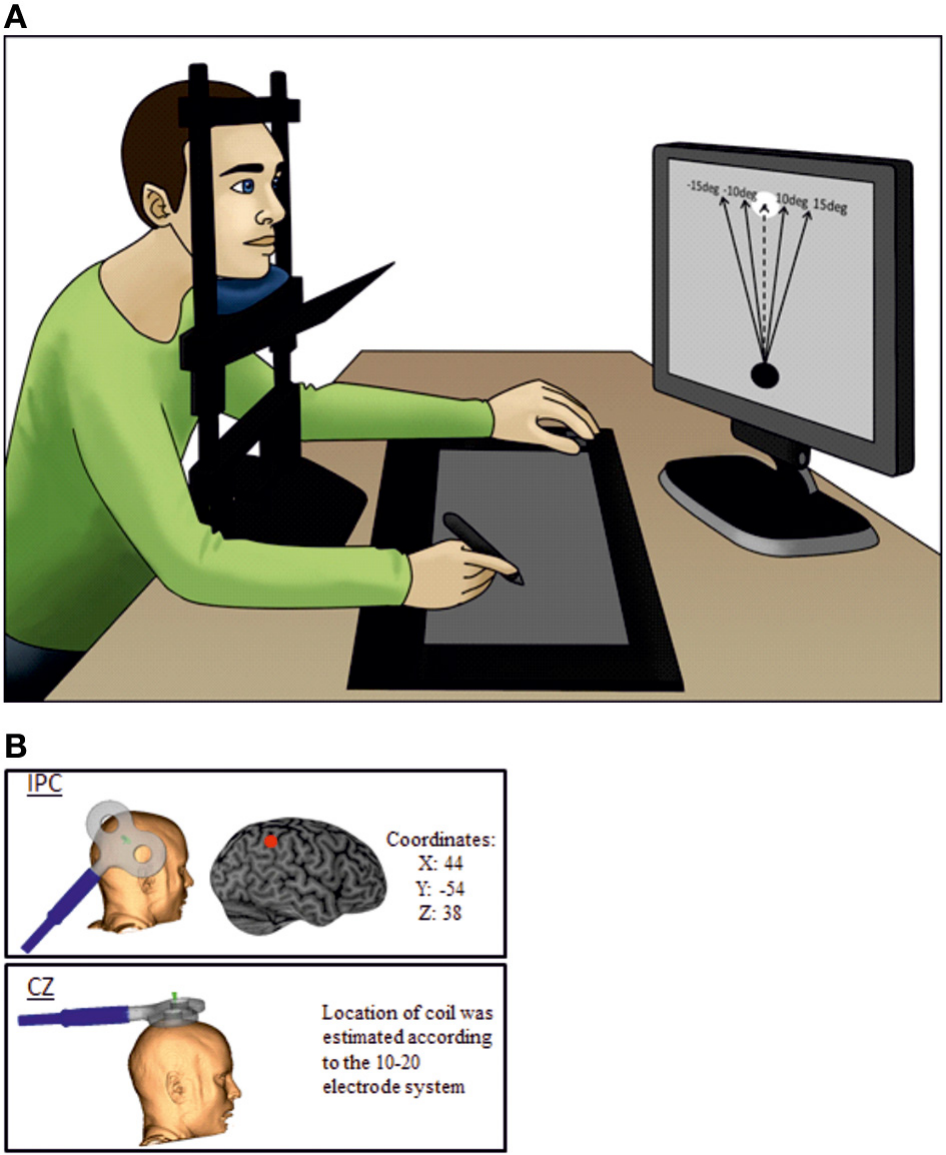
**The experimental setup. (A)** The subject is not able to see his own arms as vision is blocked. The dotted line is representing the self-controlled movement where subjects have full control of the object and the black lines represent the possible perturbations. During the experiment there were no visible lines or text on the display screen. **(B)** Illustrate coil orientation and placement.

Subjects were seated comfortably in a chair with their head resting in a chin rest 55 cm in front of a computer screen and vision of the arms blocked by a blind (see Figure 1A), so they were not able to see the digital tablet placed in front of them. On the screen, participants could see a target in the upper center and a circle in the lower center of the screen. The task was to move the circle toward the target by placing a digital pen on the tablet. As subjects did not see their own movement on the tablet, they received visual feedback about their movement from the trajectory of the circle on the screen. In two-thirds of all trials, the circle was manipulated to deviate by 10, −10, 15, and −15° away from the target regardless of the movement of the subject. The manipulations were intermingled with movements which were completely controlled by the subject (self-controlled movement). After each finished movement subjects were asked to make a quick intuitive decision whether they felt being responsible for the observed movement or if they thought the circle was externally manipulated. This decision was communicated by pressing one of two buttons with the left hand. A total number of 120 trials (80 trials evenly divided between the computer deviations and 40 self-controlled movements) were performed per session.

The paradigm design was presented in a validated custom-made program (using F#) (Ritterband-Rosenbaum et al., 2011). In accordance with the TMS safety guidelines (Rossi et al., 2009) inter-trial intervals of 3 s were added to the original paradigm to ensure sufficient breaks between rTMS trains. The sizes of the screen and tablet were 380 × 303 mm (with a resolution of 1280 × 1024 pix) and 310 × 238 mm, respectively (Tablet: Wacom, Intuos 3, Krefeld, Germany http://www.wacom.com/en/de/). Subjects were instructed to move the circle by straight, fast movements. The size of the circle and the target was 3.8 × 3.6 cm (120 × 116 pix) resulting in an actual movement distance of approximately 15 cm, which could be achieved without moving the head or torso. Three successive sessions (TMS on active site (IPC), TMS on control site (CZ) and noTMS) were conducted with 1 h of break in between. The break was added to avoid any carry-over effect of the TMS stimulation and sessions were randomized. Prior to each session, subjects were given a short introduction to familiarize themselves with the task and the TMS. For the noTMS session we placed the TMS coil in close proximity (approximately 25 cm away from the subjects' right side of the head) to the subject to keep the auditory input constant. This baseline control was chosen to confirm that TMS over CZ did not affect agency experience. Participants in the noTMS condition were aware that no direct stimulation was given; this allowed us to verify that behavior during control stimulation over CZ was not influenced by either diffuse general effects of stimulation (e.g., stimulation sensation, placebo) or by a stimulation specific effect. Since we did not expect any effect of CZ stimulation, differentiation between diffuse and specific effects in this condition was not the focus of the experimental design.

### TMS stimulation

Neuronavigation (Brainsight, Magstim Ltd) was used for precise positioning of the coil. Magnetic resonance imaging (MRI) data specific to each participant were used to ensure correct placement of the coil. Each individual MRI was normalized onto the Montreal Neurological Institute (MNI) brain template from the Brainsight software. The IPC location was found using the MNI coordinates: 44,-54, 38 (Farrer et al., 2008), whereas the control site was CZ measured by the 10–20 electrode system (Herwig et al., 2003).

Magnetic stimulation was delivered using a custom-made figure of-eight coil (external diameter of coil wing: 115 mm), connected to a MagstimRapid stimulator (Magstim Ltd, Whitland, Dyfed, UK). 1 s trains of 10 Hz TMS were given for each individual trial. This frequency was chosen because it has proven effective in modulating cognitive functions in a wide range of previous studies (Devlin et al., 2003; Leyman et al., 2009; Manenti et al., 2010; Acheson et al., 2011). The stimulation started 500 ms after the pen was placed on the tablet on top of the visual object and participants were instructed and trained to start the movement as soon as the stimulation started. Hence TMS stimulation stopped 1500 ms after the pen was placed on the tablet exceeding average trial time (mean: 643 ± 216 ms). The intensity of the TMS was set to 120% of resting motor threshold of the first dorsal interosseous muscle (FDI) (Rossini et al., 1994). During the experiment the coil was kept in position by a TMS-holder (see Figure 1B) and continuously monitored by neuronavigation.

### Data analysis

All analysis was done off-line after the experiment using Excel, SigmaPlot 12 (Systat Software Inc) and Matlab R2012a (MathWorks, Natics, MA, USA). All X,Y-coordinates from the pen on the tablet and the object on the screen were combined in order to extrapolate data from each completed trial. Agency scores and kinematic data were calculated as follows:

1. Subjective agency score (%) was calculated from the participants reporting of whether they experienced being responsible for the observed movement trajectory. A higher score corresponds to a higher rate of reporting “No, I was not responsible for the observed movement.”
2. Line curvature (mm^−1^) referred to how much the movement of the pen on the tablet deviated from the direct straight line from initial object position to the target. The smaller the value the more straight the subjects made the movement. The equation for the values is as follow:
  $$c=\frac{x^{\prime }y^{\prime \prime }-y^{\prime }x^{\prime \prime }}{{\left({x^{\prime }}^{2}+{y^{\prime }}^{2}\right)}^{3/2}}$$
3. Hit distance (mm) is the difference between the target and the end position of the pen on the tablet, i.e., the difference between the coordinates of X_pen_ and X_center of target_. Absolute values were used to evaluate the distance independent of which side of the target was hit.
4. Movement time (ms) corresponded to the time for moving the visual object to the target.
5. Answer time (ms) corresponded to the time it took subjects to decide about their agency after the movement was completed.

### Excluded data

Trials where the answer time was longer than 2 s or where the whole trial time was over 3 s were excluded from further analysis (30 trials). Additionally, trials were excluded if the curvature was more than 2 SD above that of the participant's average curvature within the same manipulation group (56 trials). In total, less than 2% of all trials were excluded.

For the kinematic data we focused the analysis on an area above and below a horizontal level of 20% from the bottom and top of the screen. The included area covered the top of the object to the bottom of the target. The cutoff meant that contaminated data which could derive from picking up the visual object or when hitting the horizontal level of the target would not affect the active cursor movement.

### Statistics

All data were checked for normality distribution and equal variance using the Shapiro-Wilk test. To test for changes in agency attribution and kinematic measures after TMS stimulation repeated-measures ANOVAS were done. In a first step a repeated measures ANOVA (rmANOVA) with the factors Site (IPS, CZ, and noTMS) and Perturbation (no perturbation, 10 and 15°) was run. In a second step, agency scores were analyzed in separate rmANOVAs for each level of perturbation (self-controlled movement, 10 and 15°). We chose to investigate conditions separately for two reasons: first, the SoA ratings reflect antagonistically on correct agency detection in non-perturbed and perturbed trials (e.g., in unperturbed trials high “self” ratings reflect good performance, in perturbed trials low “self” ratings reflect good performance). Second, the state-dependency of TMS predicts that high activity levels can “protect” from the effects of TMS (Silvanto and Pascual-Leone, 2008). This is why we hypothesized that the self-controlled movements might be most susceptible to rTMS. For those movements where a significant effect of TMS was observed, differences in kinematic parameters (curvature, hit distance, movement time) and answer time depending on TMS *and* agency attribution were explored. Only 8 participants were included in this analysis since the other participants did not have both answer types (yes/no) for some of the conditions. All *Post-hoc* comparisons were done using Holm-Sidak corrected *t*-tests. All Statistical analysis was done in SigmaPlot 12.

## Results

**Figure 2:**
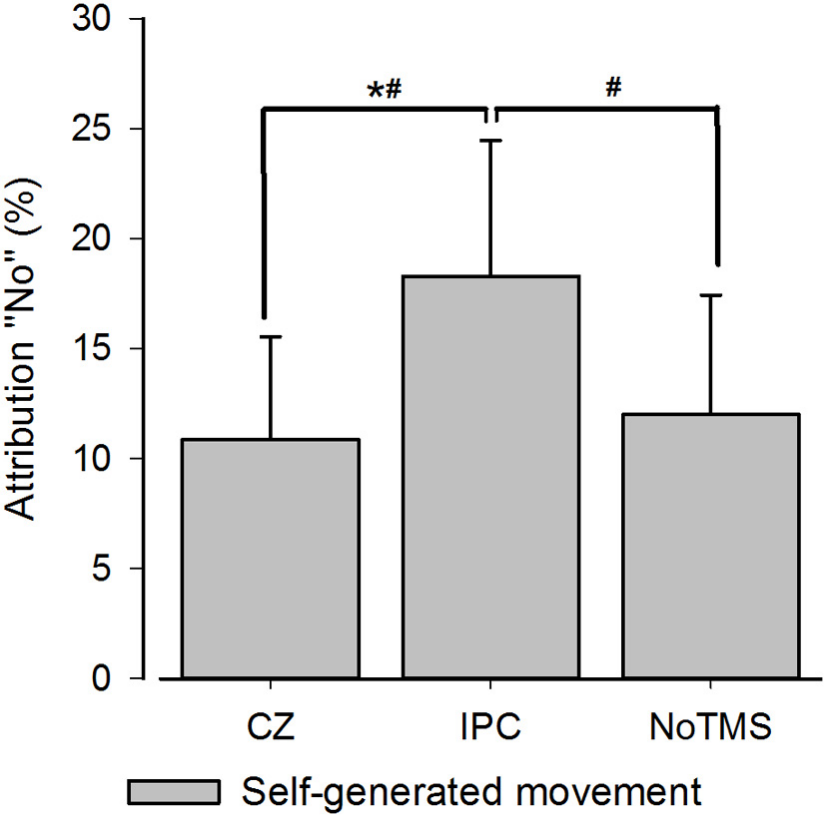
**Group averages for SoA for self-controlled movements**. The figure displays the group averaged level of agency rejection in percentage for self-controlled movements. The ^*^ indicates a significant difference when corrected for Holm-Sidak *post-hoc* test. The # identifies a significant *p*-value prior to the Holm-Sidak correction. Error bars depict inter-subject s.e.m.

**Figure 3:**
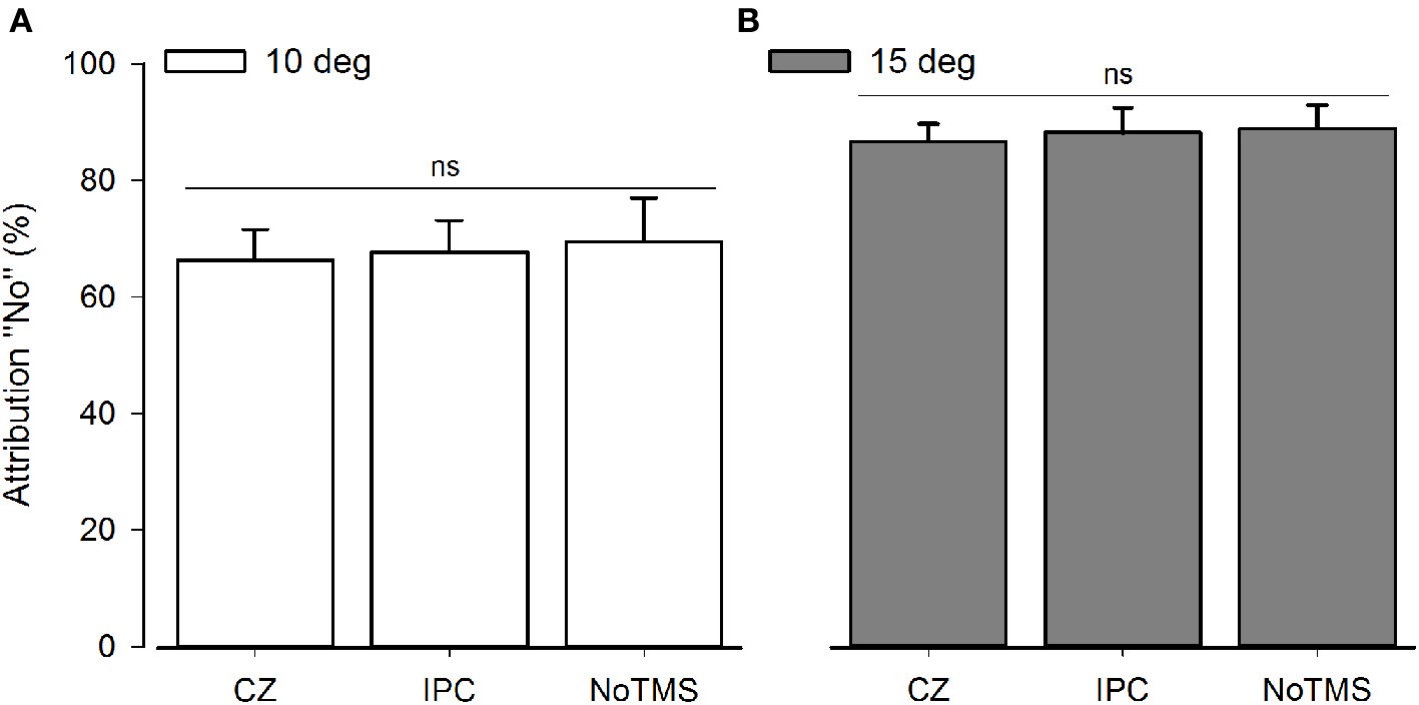
**Group average for SoA for computer-manipulated movements**. The graphs display the agency rejection for computer-manipulated movements. NS, Non-significant. **(A)** Shows 10° perturbations. **(B)** Shows 15° perturbation. The error bars depict the inter-subject s.e.m.

In the 3 × 3 rmANOVA including all levels of perturbation only a main effect for perturbation could be detected [*F*_(2)_ = 64.95; *P* < 0.001] neither the main effect for Stimulation Site [*F*_(2)_ = 0.289; *P* = 0.75] or the interaction between stimulation site and deviation reached significance [*F*_(2)_ = 0.558; *P* = 0.75]. When running a separate analysis for each perturbation level, a main effect for TMS [*F*_(2)_ = 4.62; *P* = 0.02] was detected for unperturbed movements (see Figure 2). *Post-hoc* testing confirmed higher rates of agency rejection for IPC stimulation compared to CZ stimulation and no TMS (*P* = 0.007 and *P* = 0.045, respectively) and no difference in agency rejection between CZ and no TMS (*P* = 0.421). Only the IPC-CZ comparison remained significant following Holm-Sidak correction for multiple comparisons. For perturbed movements no significant main effects was found ([*F*_(2)_ = 0.18; *P* = 0.83] and [*F*_(2)_ = 0.38; *P* = 0.68] respectively for the 10 and 15° perturbations) (see Figure 3).

We did not find any statistical differences between the different TMS sessions for the self-controlled movements when dividing the data according to the subjective reporting for Curvature_Self_ [*F*_(2)_ = 1.278; *P* > 0.5], Movement time_Self_[*F*_(2)_ = 0.712; *P* > 0.5], Answer time_Self_[*F*_(2)_ = 1.06; *P* > 0.3] or Hit distance_Self_ ([*F*_(2)_ = 0.901; *P* > 0.4]). However, for Hit distance_Self_ we were able to detect a significant main effect of assessment (yes/no) ([*F*_(1)_ = 11.67; *P* = 0.01]) indicating that the hit distance was smaller when subjects attributed the movement to themselves. None of the other variables (Hit distance_Manipulated_, Curvature_Manipulated_, Movement time_Manipulated_,or Answer time_Manipulated_) showed a significant assessment effect.

Table 1 illustrates the averaged kinematics (curvature, hit distance and movement time) and answer time for self-controlled movement in the three individual sessions. The table only contains data from eight subjects, as four subjects did not have both Yes and No assessments. Variation is depicted as 1 inter-subjects SD.

**Table 1:** **Kinematic**.

**Table for kinematic (self−controlled movements)**						
	**CZ**		**No TMS**		**IPC**	
	**Yes**	**No**	**Yes**	**No**	**Yes**	**No**
Curvature (mm^−1^)	0.046 ± 0.032	0.047 ± 0.032	0.038 ± 0.018	0.046 ± 0.032	0.039 ± 0.025	0.042 ± 0.025
Hit distance (mm)	−2.8 ± 10.6	−8.6 ± 16.7	−4.8 ± 9.3	−8.0 ± 10.6	−5.9 ± 12.0	−11.2 ± 13.6
Movement time (ms)	200.5 ± 65.3	213.0 ± 71.6	200.5 ± 60.3	205.1 ± 59.5	197.3 ± 45.7	206.4 ± 53.5
Answer time (ms)	376.4 ± 105.6	421.9 ± 197	405.5 ± 203.9	529.8 ± 241.9	404.7 ± 203.4	493.8 ± 277.4

## Discussion

When rTMS was applied over the IPC subjects were more likely to reject agency for unperturbed movements than when rTMS was given over a control site. Rejection rate for these movements increased from around 11 to 19% after IPC stimulation, and the same pattern was observed when comparing IPC stimulation with noTMS. The sense of agency for the externally perturbed movements was unaffected by IPC stimulation. The observed effect was only significant when analyzing the perturbed and unperturbed movements separately. We argue that separating movements is appropriate for two reasons: first, the SoA ratings reflect antagonistically on correct agency detection in non-perturbed and perturbed trials and errors in agency detection reflect antagonistically on attribution-errors: in the self-controlled movements participants commit errors of under-attribution whereas in the perturbed movements the participant commits errors of over-attribution. Second, we specifically hypothesized that rTMS influences the self-controlled trials more due to the state-dependency of brain stimulation (Silvanto and Pascual-Leone, 2008; Silvanto et al., 2008).

Our data for the non-perturbed movements are in line with results from Preston and Newport (2008) reporting decrease in agency for self-controlled movements after right IPC stimulation. Preston et al. also reported changes in agency perception for computer manipulated movements but it has to be noted that these changes were not significantly greater than the difference induced by TMS over a control site (Preston and Newport, 2008) suggesting that, as in our study, only the effects observed during own movements were truly site specific. In our study, *post-hoc* testing showed that increases in agency rejection were significant between the control site and the IPC and a similar pattern was found when comparing IPC and no TMS (significant when uncorrected). It is worth noting, that the significant difference between the IPC and control site was not driven by a TMS induced change in the control region since no difference could be detected between the control region and the stimulation free condition. Our data indicates that TMS over the IPC does not result in a non-specific tendency to reject agency or misattribute the observed movements across different levels of perturbations. Rather it selectively affects conditions where the feeling of agency is very high.

Comparator Models (CM) (Wolpert et al., 1995; Frith et al., 2000) have often been proposed as the underlying mechanisms of agency attribution, and can help to explain why the shift in agency perception was only observed where the feeling of agency was high (self-controlled movements). According to the CM, every movement outcome is compared to an “internal model” of the movement, which consists of the movement intention and a prediction about the movement outcome. If the error between the internal model and the actual outcome of the movement is low we perceive agency. In the case of the 10 and 15° perturbations the error between the predicted and the actual sensory feedback is high causing participants to reject agency for these movements. Potentially, IPC stimulation is not able to increase the mismatch between the predicted and observed movement for the movement types of 10 and 15° computer manipulations, and hence does not further impact agency judgments. On the other hand, for the self-controlled movements the error signal is usually small and stimulating the right IPC adds significant noise to the comparison, which creates difficulties for the subjects when determining movement agency. This could lead to higher rates of misattribution (increased agency rejection) when compared to baseline. It is probable that very challenging perturbations, with only minimal prediction-outcome errors would show significant agency alterations after interfering with normal activity in right IPC with TMS.

The IPC and the surrounding area has also been implicated in many aspects of visually guided movement control (Rushworth and Taylor, 2006) and stimulation of the posterior parietal cortex can disrupt visually guided reaching movements and the ability to correct for perturbations during reaching movements (Desmurget et al., 1999; Johnson and Haggard, 2005; Chib et al., 2009; Reichenbach et al., 2011). Since neither curvature, hit distance nor movement time were significantly affected by TMS our kinematic data suggest that the change in agency attribution was not merely caused by an altered ability to control movements. We can however not exclude the possibility that TMS had a minor effect on visual movement control that was not picked up by our kinematic analysis.

We cannot determine if subjects' based their agency decision on an online sensorimotor comparison of performance and feedback or on a post-movement evaluation of the movement outcome but the difference in hit distance (end point of the movements) between accepted and rejected agency trials (irrespective of TMS) suggests that post-movement visuo-spatial cues were used by the participants to determine agency. This notion is supported by a recent EEG-study (Ritterband-Rosenbaum et al. submitted and planned to appear in this issue), showing increased parietal-prefrontal directional coupling during the agency judgment phase, after reaching movements had been concluded. These findings and the fact that the parietal lobule has been suggested to act as an interface between retrospective reflections and online sensorimotor comparisons (Jeannerod, 2009), suggests that TMS stimulation covering both, the movement and the decision phase, as applied in this study, likely yields the strongest effect on agency detection since it is able to impact both online sensory-motor comparisons and retrospective reflections.

It is interesting that imaging studies have consistently reported increased activity in the right IPC or more specifically in the right angular gyrus with higher level of feedback perturbations (Farrer and Frith, 2002; Farrer et al., 2008; Nahab et al., 2011) whereas both our study and the work of Preston and Newport (2008) suggest that IPC stimulation is most disruptive during unperturbed movements. This is in line with the state-dependent theory of rTMS effects postulating that brain stimulation effects neural populations more when their baseline activity is low.

Generally, care has to be taken when directly comparing increases in BOLD activity with behavioral performance during TMS. First of all, the exact mechanisms of online rTMS during task performance are not completely understood: the “virtual-lesion” method assumes that trains of rTMS during performance adds external noise to the stimulated area and thereby disrupt any internal processes (Pascual-Leone et al., 2000). This approach has been shown to be state-dependent with effects depending on the underlying behavioral task. Furthermore, concurrent TMS-fMRI experiments have shown that rTMS does not necessarily result in measurable changes in bold activity at the stimulated site and can show task-specific bold-effects in remote, connected brain areas (Sack et al., 2007; Heinen et al., 2011). Our results indicating that the right IPC is most vulnerable to stimulation during the self-controlled movements are in line with the idea of state-dependent brain-stimulation but it is also possible that the observed behavioral effect was caused by effects on larger parts of a fronto-parietal network.

As mentioned earlier, the IPC is part of a larger directionally specific IPC-preSMA network (Ritterband-Rosenbaum et al., submitted and planned to appear in this issue) where SoA is associated with stronger coupling from IPC to preSMA during late task phase. These inter-regional connections indicate that “self” vs. “other” attributions should not be seen purely as increased or decreased activity in single cortical areas. Rather it is the coupled activity in a specific frequency band in the network that is needed to determine sense of agency. Our rTMS-results complement the results of the EEG-study by demonstrating that stimulation of the IPC node of the parietal-premotor network alters the sensorimotor interpretation of self-controlled movements. In this context it is interesting to speculate over the different roles of the preSMA and the IPC in creating a SoA. Moore and co-workers (Moore et al., 2010) showed that disrupting the preSMA reduced the temporal link between action and effect, an implicit measure of SoA. The authors speculate that the preSMA may use motor information to generate predictions over the sensory consequences of actions. The results of the presented study and the associated EEG study are at least consistent with this idea and may suggest that the IPC feeds the sensory feedback needed for comparison to the preSMA.

Taken together, our findings suggest that interference with rTMS alters recognition of self-controlled movements in a simple drawing task. It needs to be further clarified to what extend more complex *natural* movements are being affected by external disturbance in the IPC or in the IPC-SMA network. This could help explain behavior of patients with lesions in the areas around IPC, e.g., angular gyrus, intraparietal sulcus, supramarginal gyrus etc., which also seem important for agency detection.

## Author contributions

Anina Ritterband-Rosenbaum, Anke N. Karabanov, Mark S. Christensen, and Jens Bo Nielsen designed the experiment while Anina Ritterband-Rosenbaum and Anke N. Karabanov completed the data collection, analyzed the data and wrote the manuscript. All authors approved the final version of the manuscript.

### Conflict of interest statement

The authors declare that the research was conducted in the absence of any commercial or financial relationships that could be construed as a potential conflict of interest.
